# High vs. Low Initial Steroid Dose in Autoimmune Pancreatitis: Multicenter Cohort Study on Efficacy and Diabetes Worsening

**DOI:** 10.3390/diagnostics15212719

**Published:** 2025-10-27

**Authors:** Yuhei Iwasa, Takuji Iwashita, Keisuke Iwata, Ryuichi Tezuka, Shinya Uemura, Akinori Maruta, Shota Iwata, Yosuke Ohashi, Kensaku Yoshida, Masahito Shimizu

**Affiliations:** 1 Department of Gastroenterology, Gifu Municipal Hospital, Gifu 500-8513, Japan; festinalenteyu@gmail.com (Y.I.); tez1101@gmail.com (R.T.); 2 First Department of Internal Medicine, Gifu University Hospital, Gifu 501-1194, Japan; ueshin550621@gmail.com (S.U.); mrak5844@yahoo.co.jp (A.M.); iwthalop20@gmail.com (S.I.); yosuke-ohashi14@hotmail.com (Y.O.); shimim-gif@umin.ac.jp (M.S.); 3 Department of Gastroenterology, Shiga University of Medical Science Hospital, Setatsukinowa-cho, Otsu 520-2192, Japan; 4 Gifu Prefectural General Medical Center, Department of Gastroenterology, Gifu 500-8717, Japan; kensakuyoshidaky@gmail.com

**Keywords:** autoimmune pancreatitis, diabetes mellitus, steroid, relapse

## Abstract

**Background:** Steroid therapy is the first-line treatment for autoimmune pancreatitis (AIP) with high response rates, although steroids can cause adverse events (AEs), such as diabetes mellitus (DM). The optimal initial steroid dose has not been well studied, and initiating high-dose steroid treatment may cause worsening of DM. **Aims:** The aim of this study was to evaluate the effect of the initial steroid dosage on treatment efficacy and diabetes mellitus in the management of autoimmune pancreatitis. **Methods:** A total of 81 AIP patients treated with steroids were divided into two groups based on the starting steroid dosage: a high-dose group (HD group; >0.4 mg/kg) and a low-dose group (LD group; ≤0.4 mg/kg). Treatment efficacy (response rate, pancreatic volume), relapse rate, and DM worsening rate were analyzed. **Results:** Among the 81 patients, 58 received HD steroids, and 23 received LD steroids. The treatment response rate was 100% in both groups (58 vs. 23, *p* = 1), and the overall relapse rate was 29% vs. 26% (17 vs. 6, *p* = 0.79), with no significant difference. At 1 year, DM worsening occurred in 50% vs. 16% (25 vs. 3, *p* = 0.007), significantly more in the HD group. The risk factors for DM worsening were starting HD steroid treatment (OR 6.52, 95% CI 1.41–30.2, *p* = 0.01) and older age (OR 1.10 per year, 95% CI 1.01–1.19, *p* = 0.03). **Conclusions:** No significant difference in treatment efficacy was found between HD and LD steroid treatment for AIP. LD treatment may prevent DM worsening.

## 1. Background

Autoimmune pancreatitis (AIP) was first proposed as a disease concept by Yoshida et al. in 1995 [1]. A serological characteristic of AIP is an elevated serum IgG4 level, observed in 84.5% of AIP patients [2]. Imaging features include localized or diffuse pancreatic enlargement on CT, as well as capsule-like structures and gradual enhancement in dynamic contrast-enhanced CT [3]. Histologically, AIP is characterized by significant fibrosis and infiltration of lymphocytes and plasma cells. Diagnosis is made based on a comprehensive evaluation of these serological, imaging, and histological findings. Some cases also present with extrapancreatic legions, such as sclerosing cholangitis, sclerosing sialadenitis, retroperitoneal fibrosis, lymphadenopathy in the abdominal or pulmonary hilum, chronic thyroiditis, and interstitial nephritis, which are now considered pancreatic lesions of IgG4-related disease [4].

AIP is clinically characterized by a marked response to steroids [5]. The efficacy of treatment with steroids was reported to be 98.6% in a 2016 survey in Japan and is considered the first choice of treatment [4]. Regarding the method of steroid administration, consensus guidelines recommend a starting dosage of 0.6–1.0 mg/kg/day for 2–4 weeks, followed by tapering over at least 12 weeks [6]. However, it has been reported that 70% of patients with AIP have diabetes mellitus (DM) [7], and a high dosage of steroids may cause adverse events such as worsening of DM. In 2024, Oberbeek et al. reported that there was no significant difference in treatment effect between AIP patients who were treated at >0.4 mg/kg and ≤0.4 mg/kg, and it is hypothesized that adequate therapeutic efficacy may be achieved even with a low starting steroid dosage.

However, there are currently few reports examining the starting steroid dosage for AIP in relation to treatment effect and adverse events. Therefore, in this study, we investigated the relationship between the starting steroid dosage, treatment effect, and the worsening of DM in patients with AIP who underwent steroid therapy.

## 2. Patients and Methods

### 2.1. Study Design

This study is a retrospective multicenter study conducted at Gifu Municipal Hospital, Gifu University Hospital, and Gifu Prefectural General Medical Center. A database analysis was conducted including all AIP patients diagnosed between April 2007 and April 2023. Patients were enrolled in this study if they met the following inclusion criteria: (1) patients who met the diagnostic criteria for AIP [4] based on blood tests, imaging studies, and histological examinations, (2) patients who underwent steroid therapy, and (3) patients whose clinical course was followed up for at least 1 year. However, patients in whom steroid therapy was used a diagnostic tool due to difficulty in distinguishing between benign and malignant conditions were excluded.

The study was conducted in accordance with the Declaration of Helsinki, and approved by the Institutional Review Board of Gifu university hospital (protocol code 2024-169, date of approval 19 September 2024). The need for written informed consent was waived due to the retrospective nature of the study. Instead, an opt-out method was employed, and information about the study was made publicly available on the institution’s website and bulletin board. Participants were given the opportunity to decline participation at any time. This study protocol was approved by the Institutional Review Board of each institution.

### 2.2. Steroid Treatment

The steroid treatment for AIP was generally performed as follows: The initial dose ranged from 20 to 40 mg/day (0.95 mg/kg/day to 0.25 mg/kg/day). The dose was tapered by 5 mg every two weeks. The maintenance dose was 5 mg/day for at least one year. However, the initial dose, tapering schedule, and maintenance dose were adjusted at the physician’s discretion. Blood tests measuring HbA1c and serum IgG4 levels were conducted every one to three months for follow-up. Additionally, a CT scan was performed one to three months after treatment initiation to assess efficacy, followed by CT or MRI every six months to one year for surveillance in all patients.

### 2.3. Study Outcomes, Definitions, and Statistical Analysis

The starting steroid dosage was categorized into a high-dose (HD; >0.4 mg/kg) group and a low-dose (LD; ≤0.4 mg/kg) group based on Oberbeek et al.’s study [8], and the study outcomes were the therapeutic efficacy, recurrence rate, and rate of worsening DM at each dosage. The decision to initiate treatment with either high or low doses was at the discretion of the attending physician.

**Figure 1:**
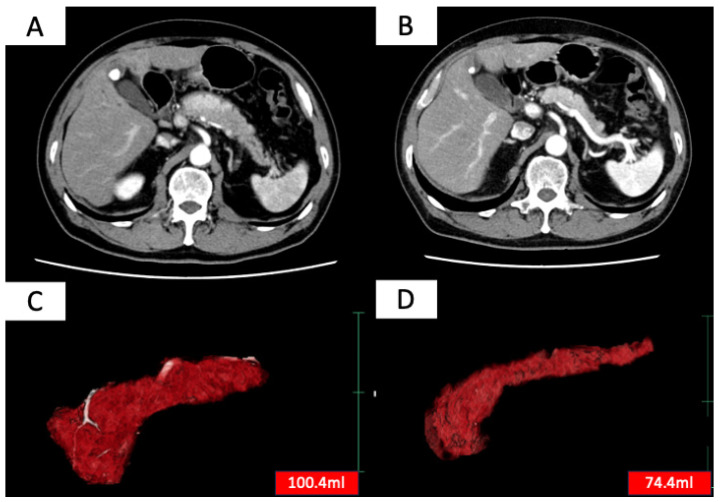
Contrast-enhanced CT images before (**A**) and after (**B**) steroid treatment, and whole pancreas images before (**C**) and after (**D**) treatment extracted using SYNAPSE VINCENT (Fujifilm, Japan). SYNAPSE VINCENT allows not only visual evaluation of the pancreas but also the measurement of pancreatic volume.

Treatment efficacy was evaluated based on two criteria: (1) improvement in pancreatic enlargement on imaging 6 months to 1 year after treatment initiation and (2) improvement in clinical symptoms. Complete remission was defined as meeting both criteria (1) and (2), and partial remission was defined as meeting either (1) or (2). Patients who achieved either complete or partial remission were considered to have responded to treatment [8]. Improvement in pancreatic enlargement was assessed by measuring pancreatic volume before treatment and 6 months to 1 year after treatment initiation using SYNAPSE VINCENT (Fujifilm, Tokyo, Japan) (Figure 1), with a reduction of more than 10% in pancreatic volume considered as an improvement.

DM worsening was defined based on a previous report [9] as follows: an increase in insulin or DM medication at 1 year after treatment initiation (or initiation of medication if previously untreated), an increase in HbA1c levels by 0.5% or more, or new onset of DM. Patients in which AIP relapsed within one year were excluded from the assessment of DM worsening. AIP relapse was defined as the recurrence of clinical symptoms or the recurrence of pancreatic enlargement or extrapancreatic lesions on imaging [10,11]. An increase in serum IgG4 level without any other clinical or radiological signs suggestive of relapse was not considered as a relapse.

Categorical or nominal variables were compared using Fisher’s exact test, and continuous variables were compared using the Mann–Whitney U test. Continuous variables are presented as median values with minimum and maximum ranges. A *p*-value of <0.05 with two-sided tests was defined as statistically significant. To identify risk factors associated with worsening DM, univariate and multivariate logistic regression analyses were performed. Variables with a *p*-value < 0.05 in the univariate analysis were further evaluated by the multivariate model. All statistical analyses were performed using EZR14 (version 1.61; Saitama Medical Center, Jichi, Japan).

## 3. Results

### 3.1. Basic Characteristics

Steroid treatment was administered to 81 patients with AIP (66 males and 15 females). The median age of the cohort was 71 years (range 27–84), and the median body mass index (BMI) was 21.8 (range 16.3–32.9). The median pre-treatment IgG4 level was 355 mg/dL (range 32–1702), and the median HbA1c was 7.0% (range 4.5–12.2). Forty-six patients had been diagnosed with DM before treatment. There were 34 patients of the diffuse type (inflammation involving two-thirds or more of the pancreas). The median initial steroid dosage was 0.49 (range 0.25–0.95). The median follow-up duration was 2019 (range 369−6319) days. Table 1 summarizes these basic characteristics.

### 3.2. Treatment Details for High Starting Dose Group and Low Starting Dose Group

We divided the patients into an HD group (>0.4 mg/kg) and an LD group (≤0.4 mg/kg) based on the starting steroid dosage. Table 2 presents the treatment details of both groups. There were 58 patients in the HD group and 23 patients in the LD group. The median tapering period of steroid was 14 weeks in the HD group and 12 weeks in the LD group, with no significant difference observed (*p* = 0.19). However, the median cumulative steroid dose at one year after the start of treatment was significantly higher in the HD group than in the LD group (2875 mg vs. 2245 mg, *p* < 0.01). Treatment efficacy was achieved in all patients in both groups (HD; 58/58, 100% vs. LD; 23/23, 100%, *p* = 1.0): complete remission was achieved in 57/58 in HD group vs. 23/23 in LD group, and partial remission was achieved in 1/58 in HD group vs. 0/23 in LD group.

**Figure 2:**
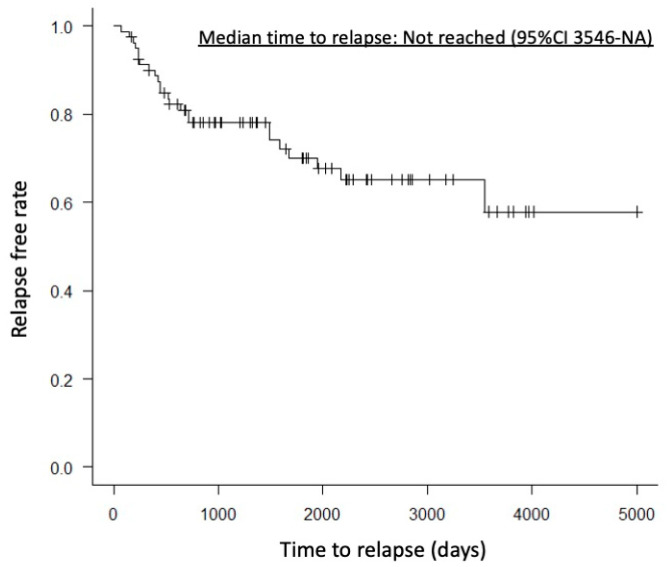
Kaplan–Meier curve showing the time to relapse. The median time to relapse was not reached (95% confidence interval, 3546–NA days).

A case with partial remission had mild pancreatic enlargement before treatment. One year after starting treatment, CT imaging showed almost no change in pancreatic volume, but the clinical symptom of abdominal pain improved. There were no significant differences between the HD and LD groups in pre-treatment pancreatic volume, pancreatic volume 6 months to 1 year after treatment, or reduction rate of pancreatic volume (76.8 mL vs. 72.5 mL, *p* = 0.71; 29.0 mL vs. 37.3 mL, *p* = 0.13; 58.0% vs. 49.5%, *p* = 0.31). AIP relapse was observed in 18 patients (29.3%) in the HD group and 6 patients (26%) in the LD group, with no significant difference (*p* = 0.79). Relapse within one year after treatment was observed in 8 patients (13.7%) in the HD group and 2 patients (8.6%) in the LD group, also showing no significant difference (*p* = 0.71). Median time to relapse was not reached (95%CI 3546-NA) (Figure 2). However, the worsening of DM was significantly more frequent in the HD group, with 25 patients (50%) compared to 3 patients (16%) in the LD group (*p* = 0.007).

### 3.3. Risk Factors of Worsening of Diabetes Mellitus

Patients were categorized into two groups based on the worsening or non-worsening of DM, and risk factors for DM worsening were investigated. Table 3 presents the patient background and treatment details for these groups. In the DM worsening group, compared to the non-worsening group, age was significantly higher (74 vs. 67 years, *p* = 0.002), there was a higher prevalence of pre-treatment DM diagnosis [21 (75%) vs. 19 (44%), *p* = 0.02], and more patients received HD therapy [25 (89%) vs. 3 (7%), *p* = 0.007]. Multivariate analysis of the risk factors for DM worsening revealed that the HD group (OR 6.52, 95% CI 1.41–30.2, *p* = 0.01) and older age (OR 1.10 per year, 95% CI 1.01–1.19, *p* = 0.03) were significant factors (Table 4).

## 4. Discussion

In this study, the effect of the initial steroid dosage on treatment efficacy and diabetes mellitus was evaluated in the management of AIP. The clinical outcomes were compared between HD (>0.4 mg/kg) and LD (≤0.4 mg/kg) initial steroid therapy. There were no significant differences in treatment efficacy or relapse rates between the two groups. However, worsening DM was more frequently observed in the HD group, and the risk factors for DM worsening in the multivariate analysis were identified as HD treatment and older age.

AIP is an autoimmune disease characterized by a marked response to steroid treatment [5]. The effectiveness of steroid treatment was reported to be 98.6% (1223/1449) in a 2016 nationwide survey in Japan [5], indicating that adequate treatment effects can be expected with steroids alone. Kamisawa et al. [12], based on the results of a multicenter study involving 563 cases from 17 facilities in Japan, demonstrated the appropriateness of a starting steroid dose of 30 mg/day (equivalent to 0.6 mg/kg), as there were no differences in remission and relapse rates between the 30 mg/day and 40 mg/day groups. As a result, the 2020 Amendment of the Japanese Consensus Guidelines for Autoimmune Pancreatitis [4] currently recommends a starting steroid dose of 0.6 mg/kg. However, initiating treatment with this relatively high dose raises concerns about an increased risk of adverse events. Notably, 43–83% of AIP patients have been reported to have concurrent DM [13,14,15,16,17], suggesting that high-dose steroid treatment may worsen or induce diabetes. Since steroid-induced DM tends to worsen with higher daily doses [18], initiating treatment with a lower dose is preferable.

Regarding the efficacy of lower doses of steroid therapy in the management of AIP, our study indicated no significant difference in treatment efficacy between the HD and LD groups (58/58, 100% vs. 23/23, *p* = 1.0). Bujis et al. conducted a retrospective analysis of patients who received steroid treatment for AIP, dividing them into low-dose (≤20 mg/day), medium-dose (30 mg/day), and high-dose (≥40 mg/day) groups, and reported no significant differences in treatment effect among these groups [19]. Furthermore, Oberbeek et al. conducted a multicenter retrospective study in Europe on the starting steroid dosage for AIP [8]. They compared the starting steroid dosage in 634 AIP patients treated with steroids across 42 facilities and found no significant difference in treatment efficacy between the groups treated with >0.4 mg/kg and those treated with ≤0.4 mg/kg (OR 0.428; 95% CI, 0.054–3.387), recommending treatment with ≤0.4 mg/kg. These results suggest that there is no difference in therapeutic effect between high-dose (>0.4 mg/kg) and low-dose (≤0.4 mg/kg), and that initial treatment at a low dose could be sufficient to achieve a therapeutic effect.

Relapse of AIP occasionally occurs after remission with steroid treatment. A 2016 national survey in Japan reported a recurrence rate of 23.4% (344/1471) among patients treated with steroids [2]. This survey compared starting steroid doses between AIP patients, and there were no significant differences in starting steroid doses between the relapse (*n* = 278; 0.548 ± 0.142 mg/kg) and non-relapse (*n* = 796; 0.544 ± 0.145 mg/kg) groups (*p* = 0.73). Similarly, our study showed no significant difference between the HD and LD groups in total relapse rate [29.3% (17/58) vs. 26.0% (6/23), *p* = 0.79] and within 1 year after treatment relapse rate [13.7% (8/58) vs. 8.6% (2/23), *p* = 0.79]. These results suggest that relapse of AIP after steroid-induced remission is not associated with the starting steroid dose. However, an association with relapse has been noted for the maintenance dose of steroids, with significantly lower relapse rates reported in patients receiving maintenance doses of ≥5 mg/day [20]. Since long-term maintenance therapy reduces the risk of recurrence, initiating treatment with a low dose is preferable to allow for gradual dose reduction. While long-term maintenance dosing decreases the risk of recurrence, increasing the cumulative dose may increase steroid-related adverse events. Further studies are needed to determine the optimal maintenance dose and duration.

In steroid treatment for AIP, the new onset or worsening of DM is one of the most common adverse events. In our current study, DM was observed in 56% (40/71) of patients prior to starting steroid treatment. Following steroid therapy, 53% (21/40) experienced worsening of DM, and while 44% (31/71) initially did not have DM, 22% (7/31) developed DM after starting steroid treatment. Overall, the incidence of new-onset or worsening of DM was 39% (28/71). In the study by Harai et al. on factors influencing glycemic control in AIP [9], 27% (9/33) of AIP patients had DM prior to steroid treatment, and after steroid treatment, 39% (13/33) had worsening DM. These results are consistent with our findings, highlighting the need to monitor for new onset or worsening of DM during steroid treatment for AIP. When comparing HD and low-dose LD steroids in relation to DM worsening, our study found a significantly higher rate of DM worsening in the HD group [50% (25/50) vs. 14% (3/21), *p* = 0.007]. Multivariate analysis of risk factors for DM worsening and onset identified the starting steroid with HD (OR 6.52, 95% CI 1.41–30.2, *p* = 0.01) and advanced age (OR 1.10 per year, 95% CI 1.01–1.19, *p* = 0.03) as significant factors. Several studies have reported that advanced age is a risk factor for steroid-induced diabetes [21,22], but to the best of our knowledge, no reports have specifically addressed the relationship between steroid dose at the start of treatment and DM in AIP. As a general rule, the risk of steroid-induced diabetes increases with the total daily dose of steroids [18]. In our study, while the initial steroid dose was identified as a risk factor, the cumulative dose was not associated with worsening of diabetes. Nevertheless, a previous study [23] reported that the incidence of steroid-induced diabetes increases with increasing cumulative steroid doses. Therefore, to reduce the risk of DM onset and worsening, it is preferable to initiate treatment with LD.

Steroid treatment is known to potentially cause various adverse events, including not only DM but also infections and osteonecrosis. In our study, we did not observe any serious adverse events other than steroid-induced DM. Regarding infections, a representative adverse event, it has been reported that the risk of administering 30 mg/day for one month is equivalent to the risk of administering 5 mg/day for three years [24]. Regarding femoral head necrosis, it has been reported that patients with a cumulative steroid dose exceeding 10,000 mg had a significantly higher incidence of femoral head necrosis compared to those with lower doses (OR 9.1; 95% confidence interval, 4.6–19.8), and an increase of 10 mg in the daily dose increased the risk of femoral head necrosis by 3.6% [25]. In the treatment of AIP, Kubota et al. [20] investigated cases with serious adverse events due to steroid treatment and found that cases with serious adverse events had significantly higher total doses compared to those without (11,307.8 vs. 6139.7 mg; *p*= 0.001), with a cut-off value calculated at 6405 mg using ROC analysis. Our study showed that the cumulative steroid dose at one year was significantly lower in the LD group compared with the HD group, which could potentially reduce the risk of steroid-induced AEs, although long-term outcomes beyond one year were not evaluated in this study. Given that the risk of adverse events, including DM, infections, and osteonecrosis, increases with higher daily and cumulative doses, it is preferable to initiate treatment with LD steroids, if possible.

### 4.1. Strengths

This study is considered a promising one, suggesting that low-dose initiation of steroid therapy in the management of AIP could reduce steroid-related adverse events without compromising treatment efficacy.

### 4.2. Limitations

This study had several limitations. This study had a retrospective design and a small cohort size, and some significant standardized mean differences were observed in patient characteristics between the HD and LD groups, indicating bias in patient selection. Additionally, the study was conducted at only three centers, which raises concerns regarding its external validity. Since multivariate analysis for relapse was not conducted in this study, the analysis of relapse cannot be considered comprehensive.

## 5. Conclusions

In the management of AIP, starting LD steroid treatment has shown no difference in treatment efficacy or relapse rates compared to HD steroid treatment, and it may help prevent the worsening of DM. However, research on starting LD steroid treatment and its long-term outcomes is still limited, necessitating further large-scale comparative studies with longer follow-up periods or prospective studies.

## Figures and Tables

**Table 1:** Basic characteristics for high starting dose group and low starting dose group.

	Total	PSL > 0.4 mg/kg	PSL ≤ 0.4 mg/kg	*p* Value	SMD
Total, *n*	81	58	23	-	-
Age, years, median (range)	70 (27–84)	72 (27–83)	67 (49–84)	0.28	0.12
sex (male/female), *n*	66/15	45/13	21/2	0.21	0.48
BMI, kg/m^2^, median (range)	21.8 (16.3–32.9)	21.4 (16.3–28.8)	22.6 (16.7–32.9)	0.09	0.17
IgG4 level before treatment, mg/dL, median (range)	355 (32–1702)	285 (32–1702)	451 (71.1–1340)	0.28	0.23
HbA1c level before treatment, %, median (range)	7.0 (4.5–12.2)	7.0 (4.5–12.2)	6.8 (5.3–11.1)	0.46	0.37
Diagnosed as DM before treatment, *n* (%)	46 (57)	34 (59)	12 (52)	0.62	0.19
Diffuse type, *n* (%)	34 (42)	26 (45)	8 (35)	0.46	0.20
Existence of IgG4 sclerotic cholangitis, *n* (%)	25 (31)	21 (36)	4 (17)	0.11	0.57
Existence of extrapancreatic lesion, *n* (%)	35 (43)	28 (48)	7 (30)	0.23	0.36
Obstructive jaundice, *n* (%)	20 (25)	16 (28)	4 (17)	0.50	0.24
Follow-up duration, days (range)	2019 (369–6319)	-	-	-	-

BMI, body mass index; DM, diabetes mellitus.

**Table 2:** Treatment details for high starting dose group and low starting dose group.

	Total	PSL > 0.4 mg/kg	PSL ≤ 0.4 mg/kg	*p* Value
Total, *n*	81	58	23	-
Treatment response, yes, *n* (%)	81 (100)	58 (100)	23 (100)	1
Complete remission/partial remission	80/1	57/1	23/0	1
Pancreas volume before start of treatment, mL (range)	76.1 (26.1–194)	76.8 (26.1–129.3)	72.5 (32.3–194)	0.71
Pancreas volume 0.5–1 year after start of treatment, mL (range)	33.9 (9–118)	29.0 (9.0–84.8)	37.3 (14.2–118)	0.13
Reduction rate of pancreas volume, %	54.5 (0.4–85.9)	58.0 (0.4–86.9)	49.5 (10.6–80.5)	0.31
Initial steroid dosage, mg/kg, median (range)	0.49 (0.25–0.95)	-	-	-
Tapering duration, week, median (range)	12.9 (5.0–133)	14 (5–110)	12 (5–133)	0.19
Maintenance dose, *n*				
5 mg/2.5 mg−1 mg/0 mg	65/13/3	49/8/1	16/5/2	0.30
Cumulative dose at 1 year after start of treatment, mg (range)	2875 (700–3925)	2875 (2175–3925)	2245 (700–2875)	<0.01
Relapse, *n* (%)	23 (28)	17 (29)	6 (26)	0.79
Relapse within 1 year from starting treatment, *n* (%)	10 (12)	8 (14)	2 (9)	0.71
Worsening of diabetes after treatment, *n* (%)	28 (39)	25 (50)	3 (14)	0.007
New onset of diabetes mellitus, *n*	7	7	0	-

BMI, body mass index; DM, diabetes mellitus.

**Table 3:** Details of DM worsening group and non-DM worsening group.

	DM Worsening	Non-DM Worsening	
Total, *n*	28	43	
Age, years, median (range)	74 (63–84)	67 (37–84)	0.002
sex (male/female), *n*	25/3	31/12	0.14
BMI, kg/m^2^, median (range)	21.4 (17,8–26.5)	22.6 (16.3–32.9)	0.14
IgG4 level before treatment, mg/dL, median (range)	394 (121–1099)	287 (32–1702)	0.29
HbA1c level before treatment, %, median (range)	7.0 (5.1–9.6)	6.8 (4.5–12.2)	0.60
Diagnosed as DM before treatment, *n* (%)	21 (75%)	19 (44%)	0.02
Diffuse type, *n* (%)	11 (39%)	18 (42%)	1
Starting dose > 0.4 mg/kg, *n* (%)	25 (89%)	3 (7%)	0.007
Tapering duration, week, median (range)	13 (6–110)	13 (5–133)	0.83
Maintenance dose of 5 mg, *n*	24	35	0.75
Cumulative dose at 1 year after start of treatment, mg (range)	2875 (2245–3925)	2525 (700–3925)	0.18
Pancreas volume before treatment, mL (range)	67.1 (26.1–194)	79.2 (31.8–129.3)	0.06
Pancreas volume 1 year after treatment, mL (range)	23.3 (9.0–54.7)	38.0 (14.8–118)	0.002
Reduction rate of pancreas volume, % (range)	60.2 (24.5–86.9)	48.4 (0.4–80.5)	0.04

DM, diabetes mellitus; BMI, body mass index.

**Table 4:** The multivariate analysis of risk factor of exacerbation of diabetes mellitus.

	Univariate Analysis		Multivariate Analysis	
	OR (95%CI)	*p* value	OR (95%CI)	*p* value
Age, years (Unit)	1.12 (1.04–1.2)	0.003	1.10 (1.01–1.19)	0.03
Male	3.23 (0.81–12.7)	0.09		
BMI, kg/m^2^ (Unit)	0.89 (0.75–1.05)	0.17		
IgG4 level before treatment (Unit)	1.00 (0.99–1.00)	0.51		
HbA1c level before treatment, % (Unit)	0.98 (0.71–1.35)	0.9		
Diagnosed as DM before treatment	3.63 (1.27–10.4)	0.02	2.64 (0.76–9.15)	0.13
Diffuse type	0.89 (0.34–2.37)	0.83		
Starting dose > 0.4 mg/kg	6.0 (1.57–23)	0.008	6.54 (1.42–30.1)	0.01
Tapering duration, week (Unit)	0.99 (0.97–1.02)	0.72		
Maintenance dose of 5 mg	1.51 (0.94–2.42)	0.08		
Cumulative dose at 1 year after start of treatment ≥ 2875 mg	1.95 (0.74–5.14)	0.17		
Pancreas volume before treatment, mL (Unit)	0.99 (0.97–1.00)	0.06	0.99 (0.97–1.01)	0.16

BMI, body mass index; DM, diabetes mellitus.

## Data Availability

The data presented in this study are not publicly available due to privacy or ethical restrictions.

## Abbreviations

The following abbreviations are used in this manuscript:

AIP, autoimmune pancreatitis; DM, CT, computed tomography; diabetes mellitus; MRI, magnetic resonance imaging; HD, high-dosage; LD, low-dosage; OR, odds ratio; CI, confidence interval.
